# Improvement of freezing of gait in patients with Parkinson’s disease by music exercise therapy: a study protocol for a randomized controlled trial

**DOI:** 10.1186/s13063-021-05258-w

**Published:** 2021-05-10

**Authors:** Kun-Peng Li, Zong-Lei Zhou, Ru-Zhen Zhou, Yan Zhu, Zeng-Qiao Zhang

**Affiliations:** 1 Department of Neurological Rehabilitation, Shanghai Second Rehabilitation Hospital, No. 25, Lane 860, Changjiang Road, Baoshan District, Shanghai, 200441 China; 2 West China School of Public Health, Sichuan University, Chengdu, China; 3 School of Rehabilitation Science, Shanghai University of Traditional Chinese Medicine, 1200 Cai Lun Road, Shanghai, 201203 China

**Keywords:** Music exercise therapy, Freezing of gait, Motor function, Parkinson’s disease

## Abstract

**Background:**

Progression of freezing of gait, a common pathological gait in Parkinson’s disease, is an important risk factor for diagnosing the disease and has been shown to predispose patients to easy falls, loss of independent living ability, and reduced quality of life. Treating Parkinson’s disease with freezing of gait is very difficult, while the use of medicine and operation has been ineffective. Music exercise therapy, which entails listening to music as you exercise, has been proposed as a treatment technology that can change patients’ behavior, emotions, and physiological activity. In recent years, music exercise therapy has been widely used in treatment of motor disorders and neurological diseases and achieved remarkable results. Results from our earlier pilot study revealed that music exercise therapy can improve the freezing of gait of Parkinson’s patients and improve their quality of life. Therefore, we aim to validate clinical efficacy of this therapy on freezing of gait of Parkinson’s patients using a larger sample size.

**Methods/design:**

This three-arm randomized controlled trial will evaluate clinical efficacy of music exercise therapy in improving the freezing of gait in Parkinson’s patients. We will recruit a total of 81 inpatients with Parkinson’s disease, who meet the trial criteria. The patients will randomly receive music exercise with and without music as well as routine rehabilitation therapies, followed by analysis of changes in their gait and limb motor function after 4 weeks of intervention. We will first use a three-dimensional gait analysis system to evaluate changes in patients’ gait, followed by assessment of their limb function, activity of daily living and fall risk.

**Discussion:**

The findings of this trial are expected to affirm the clinical application of this therapy for future management of the disease.

**Trial registration:**

Chinese Clinical Trial Registry ChiCTR1900026063. Registered on September 20, 2019

## Background and rationale

Parkinson’s disease (PD) is a common neurodegenerative disease in the elderly, which was first described by James Parkinson in 1817. The disease has an incidence of about 1–2% among people aged over 60 years old and is the second most serious neurodegenerative disease in humans [1]. Most PD patients exhibit movement or non-movement symptoms, mainly manifested in intermittent gait restriction or obstruction during walking, especially when turning, walking and avoiding obstacles, which negatively affects their quality of life [2, 3]. The current trend in aging among populations, coupled with the increasing incidence of PD, necessitates development of novel therapies for preventing, treating and delaying progression of the disease [4].

Freezing of gait (FOG) is a common type of pathological gait with disability among patients with Parkinson’s disease during mid and late stages. The condition is characterized by short-term retardation or very short pace, and usually occurs during the start of a step or change of direction [5]. Patients often describe their feet as “stuck” while trying to lift them forward, as if they were stuck to the ground. This process usually lasts for a few seconds [6]. FOG comprises three aspects, namely starting, turning, and walking freezing, and these can occur or be aggravated when a patient faces space obstacles, is under pressure, has low attention, or performs dual tasks. A previous study showed that FOG was the primary cause of walking difficulties in Parkinson’s patients and could cause falls, decreased motor function, and quality of life [7]. Modern therapies for management of Parkinson’s disease have aimed to control symptoms, reduce disability, and improve quality of life [8]. To date, the mechanism of FOG is not clear enough, and this has limited direct and effective treatment the associated symptoms, as well as development of ideal drugs and surgical treatment [9]. In fact, drug therapy has generated little effect on improvement of FOG, while advanced cases have shown very poor response to available drugs [10]. Therefore, drug therapy can only be used as an adjunctive therapy for prevention of FOG.

In the field where traditional medicine has been declared a failure, sometimes music exercise therapy can achieve curative effect. The goal of music therapy is to cause behavioral changes in patients, due to remodeling of brain nerves [11]. Rhythm is an inherent characteristic of music that can associate various behaviors with an external beat and cause synchronization of neural network behaviors [12]. Therefore, music therapists can match music with various behaviors, such as movement, phonation, respiration, and the heart rate, to cause synchronous stimulation of neurons in the brain area. These neurons are responsible for regulating the aforementioned behaviors, strengthening connection of neurons, to generate rapid and long lasting changes in patients [13].

Music intervention is used to improve socialization and cognition and emotion as well as neuromotor function, since it involves various brain regions related to emotion, motivation, cognition, and motor function [14]. In recent decades, numerous evidences have reported efficacy of music intervention, which entails passive, active, improvisational, and combined music therapy, in the clinical environment [15]. Combined music therapy is a kind of special therapy that combines music with other therapies, after integrating the characteristics of music. For example, music exercise therapy, which is a supplement to traditional exercise therapy, combines two kinds of therapies, namely exercise and music therapy. In fact, it is advantageous with regard to simplicity of operation and low cost and hence has a very broad development prospect and application value. Consequently, music exercise therapy plays an active role in treatment of various diseases and has been used to improve patients’ limb motor dysfunction and improve their quality of life [16, 17]. Previous studies have shown that cooperative dance and music training can improve the walking function of patients with Parkinson’s disease and improve their transfer ability [18–20]. However, current researches have mostly adopted a single active or passive application form of music therapy. Moreover, most of the current studies evaluating clinical efficacy of music therapy have focused on subjective indicators, such as changes in scores of the evaluation scale, while ignoring objective indicators that cause low persuasion of the research results. These facts provided rationale for the current study. In our previous study, we found that music exercise therapy could effectively improve the rehabilitation state of patients with stroke, spinal cord injury, Parkinson’s disease, and other nervous system disorders. Overall, these findings indicated that music exercise therapy does not only improve depression and rehabilitation of these patients, but also enhances their limb function, thereby improving quality of their daily life. Since this was a pilot study, it is necessary to validate these findings using a larger sample size and introduce objective observation indicators, such as three-dimensional gait analysis, to affirm the clinical efficacy of music movement therapy in improvement of FOG.

### Trial objectives

The objectives of this trial are:

1. To validate the therapeutic efficacy of music exercise therapy on FOG as well as motor function and quality of life in Parkinson’s patients.
2. To provide additional evidence on the clinical application of the treatment.

## Methods/design

### Trial design

**Fig. 1:**
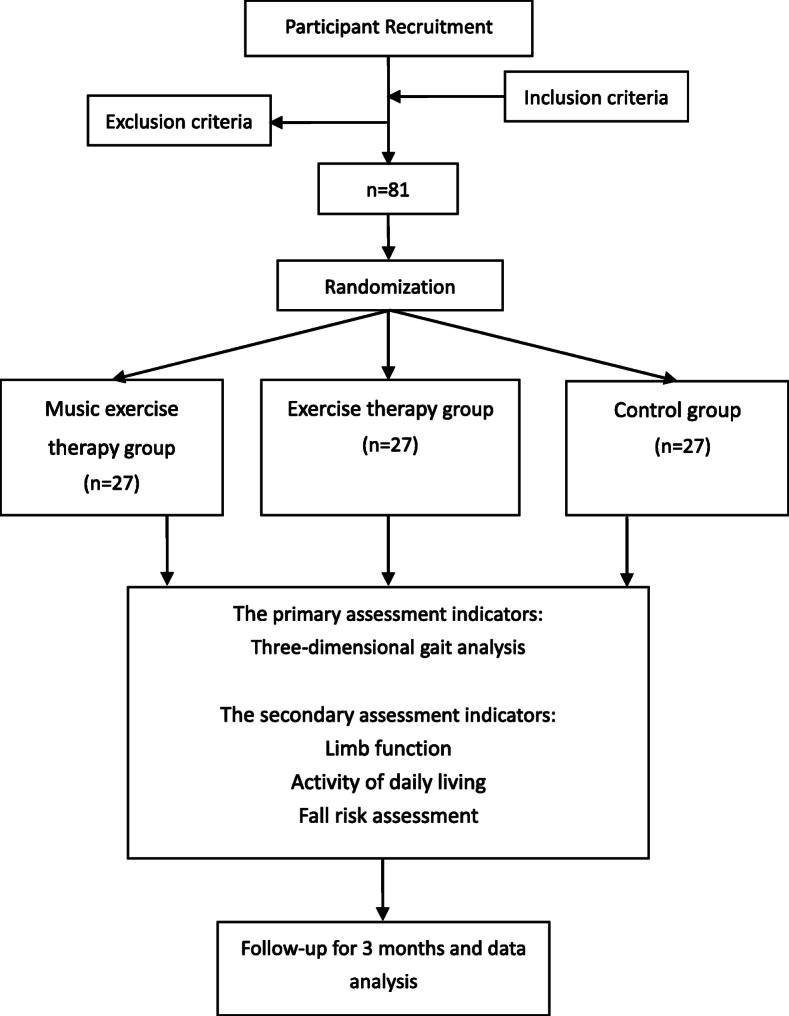
Flow chart of the study

This will be a prospective randomized controlled trial supported by Shanghai Municipal Health Commission. The trial will be carried out at the Shanghai second rehabilitation hospital. Patients who meet our pre-defined eligibility criteria will be randomly divided into three groups, namely music exercise therapy (with routine rehabilitation treatment), exercise therapy (with routine rehabilitation treatment but without music), and control group (with routine rehabilitation treatment only). Patients will be followed up for 3 months and their FOG as well as limb function observed after treatment. A flow chart of the study designed is shown in Fig. 1.

### Study setting

Research activities will be performed at the Shanghai second rehabilitation hospital, neurological rehabilitation ward.

### Eligibility criteria

#### Inclusion criteria

Participants that meet the following criteria will be included in the study: (1) clinically diagnosed with Parkinson’s disease [21]; (2) brain computed tomography (CT) and other related imaging scans confirm no brain organic disease; (3) can stand for at least 30 min, with or without auxiliary equipment, and can walk independently for 3 m or more; (4) aged between 40 and 70 years; (5) clinically manifest frozen gait, with stage 2 or 3 Hoehn and Yahr; (6) respond to levodopa or other dopaminergic therapies; (7) exhibit stable condition, clear consciousness, no aphasia, and mental retardation and can understand the content of the scale and cooperate with the examination and treatment; and (8) agree to sign an informed consent form.

### Exclusion criteria

Participants who meet the following will be excluded from the study: (1) secondary Parkinson’s disease; (2) those with deafness, aphasia, or severe cognitive impairment with difficulty to communicate normally; (3) patients with vascular dementia and frontotemporal dementia; (4) those with intolerance to standard treatment; (5) those who do not cooperate with the study program for rehabilitation; (6) those who have participated in other clinical trials within the past 3 months or are receiving other related treatments midway through the study, which may affect efficacy of this study; and (7) those with a history of neurological deficits other than Parkinson’s disease.

### Additional consent provisions for collection and use of participant data and biological specimens

Not applicable.

## Intervention

### Explanation for the choice of comparators

We hypothesize that music exercise therapy can improve the frozen gait and motor function of patients with PD.

### Intervention description

The interventions in the three groups will be as follows.

### Music exercise therapy group

In addition to routine rehabilitation treatment, we will provide music exercise therapy, in which patients will perform scheduled exercises according to the rhythm of music. This will be performed 5 times a week for 4 weeks, with 1 h each time.

#### Music selection

Music therapists will screen musical tracks and rhythms according to the actual situation and music preferences of patients with Parkinson’s disease. Thereafter, the therapists will create a personalized music playlist for each subject, because the lyrics in the music may distract attention of the PD patients, hence selection of music with lyrics will be avoided. Each playlist will be loaded into a personal music player, and subjects are allowed to choose earplugs or headphones for maximum comfort. The mode setting of the music player will be “sequential play”, and not “random play.” Moreover, music will be played by a designated music therapist, and subjects will be also told that they can request changes to their playlists at any stage during intervention.

#### Exercise therapy

While listening to music using earphones, the patients will be subjected to conduct flat start walking, turn around, and stop (at the end) trainings, as well as narrow space walking and stair step training according to the beat in the music. The patients will be expected to simultaneously complete a cycle of exercise relative to completion of the music playlist.

#### Points for attention

A precondition of the exercise therapy will be to ensure patient safety. Therefore, we will set various safety requirements with regard to the venue, patients’ clothing, and accompanying personnel, among others. Particularly, care will be taken to ensure that the site is spacious, bright, and free of obstacles, and the ground shall is flat, antiskid, and dry. Clothes and shoes will be checked to ensure that patients feel soft and comfortable during training, which is convenient for cooling and avoiding unnecessary body injury. In addition, patients will be expected to be accompanied and guarded by their families during training. Appropriate warm-up, including relatively slow and gentle exercises, will be conducted prior to the training to increase activity across all parts of the body, as well as enhance posture coordination and breathing, thereby prevent muscle straining.

### Exercise therapy group

Apart from routine rehabilitation treatment, patients in this group will be subjected to exercise therapy without music. Patients will be allowed to wear earphones without music, then subjected to flat start walking, turn around, and stop (at the end) training, as well as narrow space walking and stair step training, 5 times a week for 4 weeks, with 1 h each time.

### Control group

Participants in this group will be subjected to routine rehabilitation treatment, comprising the following: (1) routine drug treatment; (2) joint mobilization technology; (3) posture correction training; (4) balance training; (5) limb passive and active activity training; (6) walking training; (7) physical factor treatment; (8) operation ability training; and (9) daily life ability training. These will be performed 5 times a week for 4 weeks, with 1 h each time.

### Criteria for discontinuing or modifying allocated interventions

There are no special criteria for discontinuing or modifying allocated interventions.

### Strategies for improving adherence to interventions

We will adopt a variety of strategies to improve adherence to interventions. For example, we hope to establish a good relationship with the participants and fully simplify the intervention program to win their trust and cooperation. In addition, we will strengthen guidance to the participants and strive to understand and solve their problems in time to eliminate any concerns.

### Relevant concomitant care permitted or prohibited during the trial

No special provisions.

### Provisions for post-trial care

Not applicable.

### Outcome measures

Therapeutic evaluation will be carried out by the same team member who will be blinded to treatment allocation. The primary evaluation index of this study will be change of gait in PD patients, whereas the secondary evaluation indices will include evaluation of limb function, activity of daily life, and fall risk. We will adopt a three-dimensional gait analysis system for evaluation of gait information and surface electromyography for muscles related to lower limbs. We will employ the Unified Parkinson’s Disease Rating Scale (UPDRS) to assess the mental cognition, motor function, and daily living ability [22], while the Fall Efficacy Scale (FES) will be used to assess the risk of falls. Data across all measurements will be recorded in the data center [23].

### Participant timeline

**Fig. 2:**
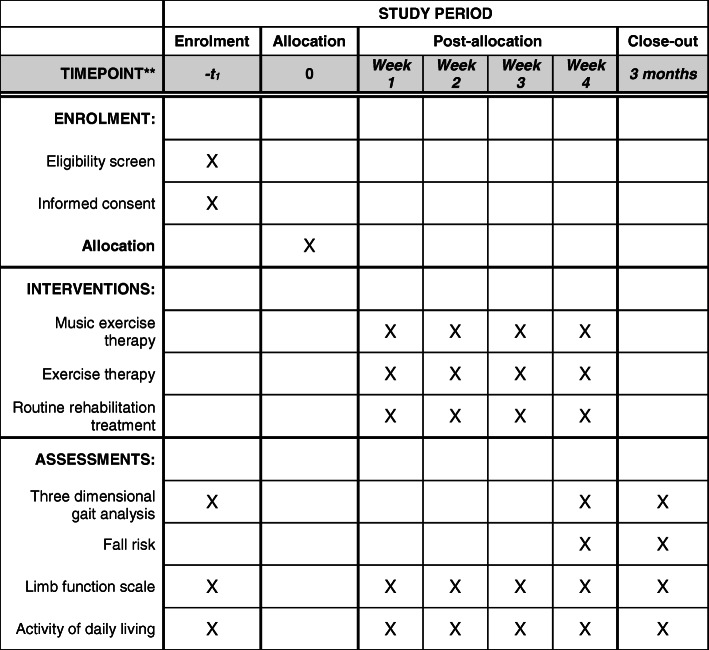
Schedule of enrolment, interventions, and assessments

Participant timeline is described in Fig. 2.

### Sample size

This will be a randomized controlled trial, seeking to evaluate efficacy of three methods for treatment of Parkinson’s FOG. The main observation index will be gait parameters. Since there is no three-dimensional gait analysis result to evaluate the effect of music exercise therapy on Parkinson’s FOG, before this study, we will temporarily use results of the unified Parkinson’s disease assessment scale (UPDRS III) from our preliminary trial for calculations. According to the pretrial, the mean value of UPDRS (III) score reduction of three groups, after treatment, is predicted to be 20.7 ± 16.6, 7.9 ± 6.3 and 2.6 ± 2.2, respectively. We will perform a two-sided test, with *α* and assurance (test efficiency) of 0.05 and 90%, respectively. According to calculations from the Pass software method (Pass15), our sample size will be 66 cases. However, we will enroll at least 81 cases, due to a 20% drop out rate.

### Recruitment and consent

Recruitment of patients began on June 1, 2020, and will be completed on December 31, 2021, or after the required number is obtained, whichever comes first. If there are not enough participants recruited by the deadline, we will submit an application for extension of time. Participant recruitment will consist of three strategies. Firstly, advertisements will be published on Shanghai Second rehabilitation hospital’s website or other online channels. Secondly, information will be shared via posters displayed in Shanghai Second rehabilitation hospital. Thirdly, recruitment posters will be distributed in nearby communities or primary clinics. Rehabilitation physicians will be requested to provide a list of individuals who meet the aforementioned inclusion criteria, while ineligible ones will be excluded. The study coordinator will invite eligible patients for the trial, by phone. Within 2 weeks of receiving the invitation, patients will receive a telephone call from a research assistant who will provide further information and ask them whether they are interested to participate. Finally, participants who provide a written informed consent and complete the baseline assessment will be randomized into the three groups and subjected to treatments.

### Consent

All participants will be required to provide a written informed consent prior to inclusion in the study. We will prepare the informed consent form, necessary audio, pictures, and other relevant materials in advance to help the participants clearly understand the purpose of the trial before recruitment. We will also explain to the participants the benefits and potential risks of participating in this study and the relevant safety measures we have put in place during the trial.

## Randomization and allocation

### Sequence generation

Randomization will be accomplished by a trained researcher, using a randomization software (Microsoft Excel) to generate a sequence of random numbers.

### Concealment mechanism

After signing the informed consent, participants will be randomly divided into the three aforementioned groups, at a ratio of 1:1:1. We will keep the allocation hidden during randomization. Slips of paper with treatment allocation will be placed in a sequentially numbered sealed opaque envelope and then opened in turn after obtaining informed consent. The research assistant will inform participants of their assigned study arm.

### Implementation

These procedures will be implemented in accordance with the protocol approved by the institutional review board (IRB). Implementation, monitoring, and incorporation of the protocol into the work arrangement will be performed by project managers. In addition, Academic Community’s Efforts to guidance will be also essential during this process.

### Blinding

The outcome assessor and data analyst will be blinded to the treatment allocation. Specifically, the assessor will enter data into a Microsoft Excel sheet, after completion of data collection, without knowledge of treatment group assignments. All entered data will be assigned corresponding codes and cleared of unnecessary information. Blinding will be broken after the data has been analyzed.

## Data collection and management

### Plans for assessment and collection of outcomes

All information shall be truthfully and accurately recorded in case report forms (CRF), at baseline and at 4 weeks after intervention. The evaluation of three-dimensional gait analysis and UPDRS will be conducted by a specially-trained evaluator, who is not clear about the trial group. The CRF and trial summary shall be submitted in time, at the end of the trial, to facilitate completion and accuracy of CRF examination.

### Plans to promote participant retention and complete follow-up

There are no specific plans to promote participant retention.

### Database management and quality control

Our research team will take effective measures to ensure the quality of research. Particularly, we will adopt the double entry method in order to ensure quality of data. The statistician will compare the two databases through the program, and in case of inconsistencies in the input results, he/she will check to identify the error. After final confirmation, the database will be saved and kept by a special person. Any future changes to the database shall be agreed upon, in writing, by the clinical study director, statistician, and data manager.

### Confidentiality

A specially assigned person will manage the relevant data during the trial period. All data will be identified using participants’ numbers, which will not directly display their personal information, to ensure confidentiality. Data will not be shared unless, explicitly approved by the researchers.

### Plans for collection, laboratory evaluation, and storage of biological specimens for genetic or molecular analysis during the trial period of for future use

None.

### Data access

Access to the data will be via contact to the corresponding author, upon signing of an agreement.

### Data analysis

All data analyses will be detailed in a Statistical Analysis Plan, to be agreed upon prior to database locking and release of intervention allocations. This will be after a thorough quality control check on all obtained datasets. Statisticians will use statistical product and service solutions (SPSS) or statistical analysis system (SAS) for statistical analyses, according to the intention-to-treat principle.

### Analysis of primary and secondary outcome measures

Analysis of categorical variables will be performed using the Pearson’s *χ*^2^ or Fisher’s exact tests, whereas continuous variables will be analyzed using a Student’s *t* test or appropriate non-parametric methods. All datasets will be analyzed at 95% confidence interval and will be tested by double-sided statistics. Data will be expressed as means ± standard deviation (SD). Data will first be tested for normality and homogeneity of variance prior to analysis, and if the normal distribution is satisfied, a *t* test will be used to compare 2 groups, whereas analysis of variance (ANOVA) with LSD or SNK methods will be used for multiple comparisons. Datasets that are not normally distributed will be analyzed using a rank sum test.

### Interim analyses

No interim analyses are planned.

### Methods for additional analyses (e.g., subgroup analyses)

Not applicable.

### Methods to handle protocol non-adherence and missing data

Analyses will be performed according to the intention-to-treat principle.

### Plans to give access to the full protocol, participant-level data, and statistical code

The full protocol, participant-level data, and statistical code will be available from the corresponding author, upon reasonable request.

## Oversight and monitoring

### Composition of the coordinating center and trial steering committee

The Trial Steering Committee (TSC) will comprise chief principals, investigators, a statistician, and trial manager.

### Composition of the data monitoring committee, its role and reporting structure

The TSC will be in charge of data monitoring.

### Adverse event (AE) reporting and harms

Any adverse medical events to the participants will be collected after the subjects have signed the informed consent and agreed to participate in the study. Events will not be considered to be related to music exercise therapy if they occur after the participant has signed the informed consent, but before the intervention. All adverse events occurring after enrolment into the study and before discharge will be recorded and reported to the local Institutional Review Board.

### Frequency and plans for auditing trial conduct

We will conduct systematic and independent supervision on trial-related activities and documents, to determine whether implementation of the trial as well as data recording, analysis, and reporting are in line with the trial protocol, the sponsor’s standard operating procedures (SOP), good clinical practice (GCP), and applicable management requirements. The TSC will have biweekly meetings, to discuss and monitor progress of the trial.

### Ethics and dissemination

Any significant modifications to the trial protocol will require ethical review.

### Dissemination policy

Study findings will be widely disseminated through manuscript publication in a high-impact peer-reviewed journal.

### Plans for communicating important protocol amendments to relevant parties (e.g., trial participants and ethical committees)

All substantial protocol amendments will be made by the principal investigators, following approval from IRB and the ethics committee.

## Discussion

FOG, the most common gait abnormality that occurs during mid and late stages of Parkinson’s disease, can lead to falls and injuries. To date, drug treatment has not effectively managed the disease although rehabilitation treatment has shown significant promise. Early recognition and intervention of FOG can reduce disability rate and improve prognosis. Results from our pilot study showed that music exercise therapy can improve the FOG of Parkinson’s patients and improve their quality of life. However, its long-term effects are not known, necessitating further research. Therefore, we propose a prospective randomized clinical trial to validate the clinical efficacy of music exercise therapy for treatment of FOG in Parkinson’s patients.

To eliminate the interference of headphone wearing behavior on test results, we have proposed a set of exercises without music. Bias exists in all clinical trials. Therefore, we acknowledge that blinding music therapists and patients to the intervention will be the most challenging aspect during designing of this randomized controlled trial. Consequently, we will blind our evaluators to minimize potential bias and further adopt a three-dimensional gait analysis as part of the approach for evaluating our results. Previous studies have widely used this analysis system to evaluate gait, especially in neurological diseases. We will also apply time and space and ankle kinematic parameters as well as electrophysiological measurements during the process of walking, and this is expected to provide quantitative information about gait and reduce the interference of subjective factors in the scale evaluation. We believe that our findings will help improve FOG and motor function in patients with Parkinson’s disease.

## Trial status

The total registration period will last for 2 years, while the follow-up will be for 3 months. Patient recruitment began on January 1, 2020, and the trial is currently underway. Protocol version number and date: V1.0, May 16, 2020. Recruitment of patients is expected to be completed in December 2021.

## Data Availability

Data sharing is not applicable to this article as no datasets have so far been generated or analyzed.
